# Improved Attitude and Heading Accuracy with Double Quaternion Parameters Estimation and Magnetic Disturbance Rejection

**DOI:** 10.3390/s21165475

**Published:** 2021-08-13

**Authors:** Assefinew Wondosen, Jin-Seok Jeong, Seung-Ki Kim, Yisak Debele, Beom-Soo Kang

**Affiliations:** Department of Aerospace Engineering, Pusan National University, Busan 46241, Korea; wondebly@pusan.ac.kr (A.W.); js775@pusan.ac.kr (J.-S.J.); akkim7631@pusan.ac.kr (S.-K.K.); yisaktol@pusan.ac.kr (Y.D.)

**Keywords:** attitude, heading, extended Kalman filter, estimation, IMU sensor, magnetic disturbance rejection

## Abstract

The use of unmanned aerial vehicle (UAV) applications has grown rapidly over the past decade with the introduction of low-cost microelectromechanical system (MEMS)-based sensors that measure angular velocity, gravity, and magnetic field, which are important for an object orientation determination. However, the use of low-cost sensors has also been limited because their readings are easily distorted by unwanted internal and/or external noise signals such as environmental magnetic disturbance, which lead to errors in attitude and heading estimation results. In an extended Kalman filter (EKF) process, this study proposes a method for mitigating the effect of magnetic disturbance on attitude determination by using a double quaternion parameters for representation of orientation states, which decouples the magnetometer from attitude computation. Additionally, an online measurement error covariance matrix tuning system was implemented to reject the impact of magnetic disturbance on the heading estimation. Simulation and experimental tests were conducted to verify the performance of the proposed methods in resolving the magnetic noise effect on attitude and heading. The results showed that the proposed method performed better than complimentary, gradient descent, and single quaternion-based EKF.

## 1. Introduction

The attitude and heading reference system (AHRS) plays a significant role in navigation applications. Vehicles with any degree of navigation autonomy require an AHRS to continuously monitor their orientations with respect to a specific reference system [1]. One of the most important applications of an AHRS is the flight control of unmanned aerial vehicles (UAVs). Evidently, UAVs’ global market size has rapidly increased over the last decade owing to their intriguing applications in entertainment, transportation, rescue operations, navigation, military, and other fields. Thus, there is a growing need for an accurate and dependable AHRS.

An AHRS consists of MEMS-based tri-axis sensors, including gyroscopes, accelerometers, and magnetometers, to collect important information about the angular rotation speed, gravity, and Earth’s magnetic field, respectively. The AHRS can potentially determine the 3D orientation of a sensor device by integrating the gyroscope output from known initial conditions with gravity and magnetic field measurements from the accelerometer and magnetometer [2]. However, a precise calculation of sensor orientation is still an onerous task, and the effect of magnetic interference on the magnetometer is one of the barriers [3]. Several researchers have devised algorithms for calculating sensor orientation using low-cost microelectromechanical system (MEMS) sensors. One of the most well-known and preferred algorithms for attitude estimation is the Kalman filter. For instance, a two-step geometrically intuitive correction algorithm is combined with a quaternion-based Kalman filter to estimate attitude in real time [4]. Similarly, a quaternion-based Kalman filter with an adaptive-step gradient descent algorithm was presented in [5], with the goal of offsetting the effect of magnetic distortion. Both studies mentioned earlier focused on reducing magnetic disturbances to improve attitude accuracy by decoupling the attitude and heading calculation. Other studies, such as [6], also attempted to overcome the extended Kalman filter (EKF) estimation accuracy problem by varying the measurement error covariance matrix using a fuzzy-adaptive method. In other words, the device vibration, external acceleration, and magnetic disturbance were considered to make a fuzzy judgment about the selection of the measurement error covariance. In addition, the problem of finding the best measurement error covariance based on sensor data history was addressed using an analytical technique that included transform-based and learning-based approaches for determining the optimal measurement error covariance matrix [7]. In another study, Fan et al. [8] performed a thorough evaluation of various approaches used by different researchers to overcome the challenges associated with the MEMS sensors mentioned earlier, and they presented a comparative performance assessment of the approaches in readily understandable way to spot the problems easily. In particular, the study provided a performance comparison between attitude estimation and magnetic disturbance decoupling, magnetic disturbance compensation, online gyroscope bias compensation, and sensor fusion algorithms.

Poulose et al. [9] addressed five main algorithms in depth: linear Kalman filter (LKF), extended Kalman filter (EKF), unscented Kalman filter (UKF), particle filters (PF), and complementary filters (CF). Furthermore, the mathematical formulation of each algorithm was explained well, and the algorithm performances were compared. However, aside from pointing out the output differences between them, the influence of magnetic interference on attitude estimation and the adequacy of the results obtained by each algorithm were not specified clearly. To boost the attitude estimation accuracy, Farhangian et al. [10] proposed an EKF-based error prediction and PI controller system. The algorithm in this study predicts the attitude error by considering the measurement data profile of the gyroscope and uses that error as feedback for the PI controller to constructively change the determined attitude value, but the improvement is not sufficient. Youn [11] also presented a magnetometer error-tolerant method for UAV applications. This study presented a magnetometer-free AHRS during magnetometer failure. The effort put forward in this research to address the problem caused by magnetometer is commendable. However, in the absence of environmental magnetic disturbances or tamper with the magnetometer, no solution avoided the impact of an imprecise magnetometer on attitude estimation.

Despite the findings of the previously mentioned studies, some problems remain. One of the most serious concerns in the UAV industry is the lack of accurate and reliable information about attitude and heading, especially in environments where external magnetic fields may present such as in warehouses, tunnels and other indoor environments. In such an environment, the magnetometer’s reading of the Earth’s magnetic field is tempered by unexpected magnetic fields in its surroundings [12,13,14]. Therefore, this study proposes a method for avoiding the effects of magnetic disturbance on attitude estimation using double quaternion parameters estimation techniques that decouple attitude and heading calculations. In addition, the conditional magnetic disturbance due to the dynamic environment is mitigated by applying magnetic disturbance detection methods and using alternative strategies in the disturbance state. In addition, other related algorithms are thoroughly evaluated and tested for verification. The algorithm was also verified with experimentation and computer simulation.

## 2. Quaternion-Based Attitude and Heading Representation

Different methods are used to represent a rigid body orientation in three dimensions (3D). Euler used three sets of angles (roll, pitch, and yaw) to describe the definite rotation of an object frame, which is called the body frame, with respect to a given reference frame (inertial frame). Unlike the Euler representation, four parameters are used in quaternion representation for 3D rotation quantification, with the constraint that the sum of squares for each parameter equals unity [15,16,17].

**Figure 1:**
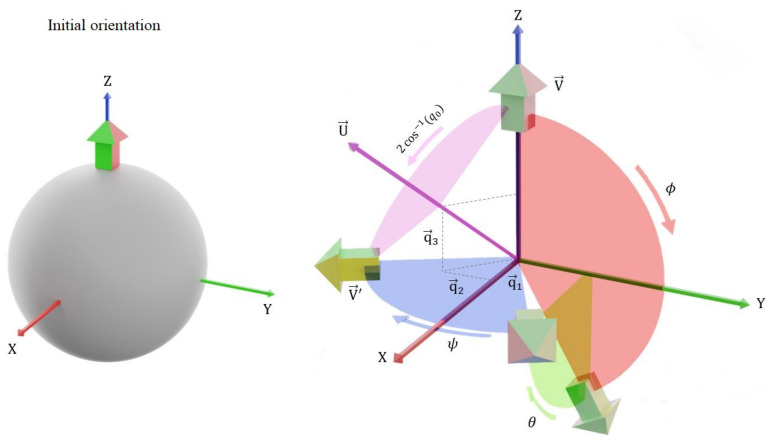
Illustration of Euler and quaternion rotations.

The rotated object final orientation is determined based on Euler angles or quaternion parameters, as given in Equations (2) and (3). Let *V* be a vector pointing to the initial front direction of the object and let the final object orientation be represented by *V*${}^{\prime }$ as shown in Figure 1. Then, *V*${}^{\prime }$ is computed from *V* and the Euler rotation angles, as indicated in Equation (2).

(1) $$V=\left[\begin{array}{c} v_{1}\\ v_{2}\\ v_{3} \end{array}\right],V^{\prime }=\left[\begin{array}{c} v_{1}^{\prime }\\ v_{2}^{\prime }\\ v_{3}^{\prime } \end{array}\right]$$

(2) $$V^{\prime }=\left[\begin{array}{ccc} cos\psi cos\theta & cos\theta sin\psi & -sin\theta \\ cos\psi sin\phi sin\theta -cos\phi sin\psi & cos\phi cos\psi +sin\phi sin\psi sin\theta & cos\theta sin\phi \\ sin\phi sin\psi +cos\phi cos\psi sin\theta & cos\phi sin\psi sin\theta -cos\psi sin\phi & cos\phi cos\theta \end{array}\right]\left[\begin{array}{c} v_{1}\\ v_{2}\\ v_{3} \end{array}\right]$$

where ϕ, θ and ψ represent the Euler rotation angles roll, pitch, and yaw, respectively, for the rotation sequence XYZ. Similarly, *V*${}^{\prime }$ is obtained from the quaternion parameters and *V*, as shown in Equation (3).

(3) $$V^{\prime }=\left[\begin{array}{ccc} q_{0}^{2}+q_{1}^{2}-q_{2}^{2}-q_{3}^{2} & 2(q_{1}q_{2}+q_{0}q_{3}) & 2(q_{1}q_{3}-q_{0}q_{2})\\ 2(q_{1}q_{2}-q_{0}q_{3}) & q_{0}^{2}-q_{1}^{2}+q_{2}^{2}-q_{3}^{2} & 2(q_{2}q_{3}+q_{0}q_{1})\\ 2(q_{1}q_{3}+q_{0}q_{2}) & 2(q_{2}q_{3}-q_{0}q_{1}) & q_{0}^{2}-q_{1}^{2}-q_{2}^{2}+q_{3}^{2} \end{array}\right]\left[\begin{array}{c} v_{1}\\ v_{2}\\ v_{3} \end{array}\right]$$

where q = $\left[\begin{array}{cccc} q_{0} & q_{1} & q_{2} & q_{3} \end{array}\right]$ is the quaternion representation of rotation.

### 2.1. Attitude and Heading Identification from Accelerometer and Magnetometer Measurements

#### 2.1.1. Finding Euler Angles

The strength of the gravitational field measured by the accelerometer along its vertical and horizontal axes can help determine the vehicle’s roll and pitch angles if the accelerometer’s and the vehicle’s axes are aligned in the same direction. The direction of gravity is always vertically downward, and its magnitude is constant. Therefore, depending on the current orientation of the sensor, the component of the gravitational field detected along the sensor axes varies, and this change is used as input to calculate the roll and pitch angles [4,12]. The acceleration components detected by the accelerometer were related to the orientation angles in Equation (4).

(4) $$\left[\begin{array}{c} a_{x}\\ a_{y}\\ a_{z} \end{array}\right]=\left[\begin{array}{ccc} cos\psi cos\theta & cos\theta sin\psi & -sin\theta \\ cos\psi sin\phi sin\theta -cos\phi sin\psi & cos\phi cos\psi +sin\phi sin\psi sin\theta & cos\theta sin\phi \\ sin\phi sin\psi +cos\phi cos\psi sin\theta & cos\phi sin\psi sin\theta -cos\psi sin\phi & cos\phi cos\theta \end{array}\right]\left(g-a_{r}\right)$$

If there is no linear acceleration (i.e., $a_{r}\approx 0$), then the normalized gravity vector is substituted by ${\left[\begin{array}{ccc} 0 & 0 & 1 \end{array}\right]}^{T}$:

(5) $$\begin{array}{cc} \frac{1}{a^{N}}\left[\begin{array}{c} a_{x}\\ a_{y}\\ a_{z} \end{array}\right]\; & =\left[\begin{array}{ccc} cos\psi cos\theta & cos\theta sin\psi & -sin\theta \\ cos\psi sin\phi sin\theta -cos\phi sin\psi & cos\phi cos\psi +sin\phi sin\psi sin\theta & cos\theta sin\phi \\ sin\phi sin\psi +cos\phi cos\psi sin\theta & cos\phi sin\psi sin\theta -cos\psi sin\phi & cos\phi cos\theta \end{array}\right]\left[\begin{array}{c} 0\\ 0\\ 1 \end{array}\right]\\ a^{N} & =\sqrt{a_{x}^{2}+a_{y}^{2}+a_{z}^{2}} \end{array}$$

Simplifying:

(6) $$\frac{1}{a^{N}}\left[\begin{array}{c} a_{x}\\ a_{y}\\ a_{z} \end{array}\right]=\left[\begin{array}{c} -sin\theta \\ cos\theta sin\phi \\ cos\phi cos\theta \end{array}\right]$$

Solving for ϕ and θ:

(7) $$\begin{array}{cc} \phi & =tan^{-1}\left(\begin{array}{c} \frac{a_{y}}{a_{z}} \end{array}\right)\\ \theta & =tan^{-1}\left(\begin{array}{c} \frac{-a_{x}}{\sqrt{a_{y}^{2}+a_{z}^{2}}} \end{array}\right) \end{array}$$

After determining the attitude angles, the heading angle was computed from the attitude and magnetometer readings [18]. The magnetometer measures the ambient magnetic field, which is a composition of the Earth’s geomagnetic field and local magnetic disturbance. We used H to represent the vector of the geomagnetic field in the north-east-down (NED) frame. Its magnitude is $H_{0}$, and its direction deviates from geographic north by the declination angle α and from the surface of the Earth by inclination angle β. It is mathematically described in Equation (8).

(8) $$H=\left[\begin{array}{c} H_{x}\\ H_{y}\\ H_{z} \end{array}\right]=H_{0}*\left[\begin{array}{c} cos\beta cos\alpha \\ cos\beta sin\alpha \\ sin\beta \end{array}\right]$$

The value of declination angle α and inclination angle β depend on geographical location. Assuming that sensor frame is rotated relative to the NED frame by the three Euler angles (ϕ,θ,ψ), one can express the geomagnetic field vector in the sensor frame as shown in Equation (9).

(9) $$\left[\begin{array}{c} m_{x}\\ m_{y}\\ m_{z} \end{array}\right]=\left[\begin{array}{ccc} cos\psi cos\theta & cos\theta sin\psi & -sin\theta \\ cos\psi sin\phi sin\theta -cos\phi sin\psi & cos\phi cos\psi +sin\phi sin\psi sin\theta & cos\theta sin\phi \\ sin\phi sin\psi +cos\phi cos\psi sin\theta & cos\phi sin\psi sin\theta -cos\psi sin\phi & cos\phi cos\theta \end{array}\right]\left[\begin{array}{c} H_{x}\\ H_{y}\\ H_{z} \end{array}\right]$$

Substituting Equation (8) into Equation (9), then normalizing and solving for the heading angle ψ,

(10) $$\begin{array}{cc} \left[\begin{array}{c} m_{x}^{N}\\ m_{y}^{N}\\ m_{z}^{N} \end{array}\right] & =\frac{1}{H_{0}}\left[\begin{array}{c} m_{x}\\ m_{y}\\ m_{z} \end{array}\right]\\ \psi & =tan^{-1}\left(\begin{array}{c} \frac{sin\phi *m_{z}^{N}-cos\phi *m_{y}^{N}}{cos\theta *m_{x}^{N}+sin\theta sin\phi *m_{y}^{N}+sin\theta cos\phi *m_{z}^{N}} \end{array}\right)+\alpha \end{array}$$

where $m_{x}^{N}$, $m_{y}^{N}$, and $m_{z}^{N}$ represent the normalized magnetometer readings of the magnetic field with respect to the sensor frame, respectively.

#### 2.1.2. Finding Quaternion Parameters

The Earth gravitational field vector measured with respect to the sensor frame and NED frame is related to the vector rotation formula presented in Equation (3). The gravity vector in the NED frame is normalized for simplicity, that is, g = ${\left[\begin{array}{ccc} 0 & 0 & 1 \end{array}\right]}^{T}$.

(11) $$\left[\begin{array}{c} a_{x}\\ a_{y}\\ a_{z} \end{array}\right]=\left[\begin{array}{ccc} q_{0}^{2}+q_{1}^{2}-q_{2}^{2}-q_{3}^{2} & 2(q_{1}q_{2}+q_{0}q_{3}) & 2(q_{1}q_{3}-q_{0}q_{2})\\ 2(q_{1}q_{2}-q_{0}q_{3}) & q_{0}^{2}-q_{1}^{2}+q_{2}^{2}-q_{3}^{2} & 2(q_{2}q_{3}+q_{0}q_{1})\\ 2(q_{1}q_{3}+q_{0}q_{2}) & 2(q_{2}q_{3}-q_{0}q_{1}) & q_{0}^{2}-q_{1}^{2}-q_{2}^{2}+q_{3}^{2} \end{array}\right]\left[\begin{array}{c} 0\\ 0\\ 1 \end{array}\right]$$

Equation (11) has infinite solutions because the number of unknown variables is greater than the number of equations. However, the gravitational vector does not provide any information about rotation around the Z-axis. Therefore, the quaternion parameter $q_{3}$ can be set to zero. Consequently, finding a finite solution for Equation (11) becomes possible. After a mathematical derivation, the quaternion parameters are represented in terms of the acceleration measured by the accelerometer. The derivation was taken from [1].

(12) $$q_{att}=\left\{\begin{array}{c} {\left[\begin{array}{cccc} \sqrt{\frac{a_{z}+1}{2}} & -\frac{a_{y}}{\sqrt{2(a_{z}+1)}} & \frac{a_{x}}{\sqrt{2(a_{z}+1)}} & 0 \end{array}\right]}^{T},a_{z}\geq 0\\ {\left[\begin{array}{cccc} -\frac{a_{y}}{\sqrt{2(1-a_{z})}} & \sqrt{\frac{1-a_{z}}{2}} & 0 & \frac{a_{x}}{\sqrt{2(1-a_{z})}} \end{array}\right]}^{T},a_{z}<0 \end{array}\right.$$

Similarly, the equation that relates the heading quaternion representation to the magnetometer readings can be derived. It is clear that the heading component of the orientation quaternion parameter is independent of any rotation about the X- and Y-axes. Therefore, the quaternion parameters representing the axis of the rotation vector should be constrained only in the vertical direction. As a result, quaternion $q_{hdg}$ has the following form:

(13) $$q_{hdg}={\left[\begin{array}{cccc} q_{0} & 0 & 0 & q_{3} \end{array}\right]}^{T}$$

The magnetic field measured by the magnetometer can be mapped to the horizontal and vertical components of the Earth’s magnetic field using Equation (14)

(14) $$\begin{array}{cc} \; & \left[\begin{array}{c} m_{x}\\ m_{y}\\ m_{z} \end{array}\right]=\left[\begin{array}{ccc} q_{0}^{2}+q_{1}^{2}-q_{2}^{2}-q_{3}^{2} & 2(q_{1}q_{2}+q_{0}q_{3}) & 2(q_{1}q_{3}-q_{0}q_{2})\\ 2(q_{1}q_{2}-q_{0}q_{3}) & q_{0}^{2}-q_{1}^{2}+q_{2}^{2}-q_{3}^{2} & 2(q_{2}q_{3}+q_{0}q_{1})\\ 2(q_{1}q_{3}+q_{0}q_{2}) & 2(q_{2}q_{3}-q_{0}q_{1}) & q_{0}^{2}-q_{1}^{2}-q_{2}^{2}+q_{3}^{2} \end{array}\right]\left[\begin{array}{c} H_{x}\\ H_{y}\\ H_{z} \end{array}\right]\\ \; & R^{T}\left(q_{hdg}\right)\left[\begin{array}{c} m_{x}\\ m_{y}\\ m_{z} \end{array}\right]=\left[\begin{array}{c} B_{h}\\ 0\\ m_{z} \end{array}\right],\;\mathrm{when}\;\mathrm{the}\;x-\mathrm{axis}\;\mathrm{of}\;\mathrm{the}\;\mathrm{sensor}\;\mathrm{is}\;\mathrm{aligned}\;\mathrm{to}\;\mathrm{the}\;\mathrm{north}\; \end{array}$$

where $B_{h}=\left(H_{x}^{2}+H_{y}^{2}\right)$. The complete solution that avoids singularity is presented next.

(15) $$q_{hdg}=\left\{\begin{array}{c} {\left[\begin{array}{cccc} \frac{\sqrt{B_{h}+m_{x}\sqrt{B_{h}}}}{\sqrt{2B_{h}}} & 0 & 0 & \frac{m_{y}}{\sqrt{2}\sqrt{B_{h}+m_{x}\sqrt{B_{h}}}} \end{array}\right]}^{T},m_{x}\geq 0\\ {\left[\begin{array}{cccc} \frac{m_{y}}{\sqrt{2}\sqrt{B_{h}-m_{x}\sqrt{B_{h}}}} & 0 & 0 & \frac{\sqrt{B_{h}-m_{x}\sqrt{B_{h}}}}{\sqrt{2B_{h}}} \end{array}\right]}^{T},m_{x}<0 \end{array}\right.$$

Finally, a single quaternion representing both attitude and heading is the product of the two quaternions $q_{attd}$ and $q_{hdg}$, as shown in Equation (16).

(16) $$q=q_{att}⊗q_{hdg}$$

### 2.2. Attitude and Heading Computation from Gyroscope Reading

The gyroscope measures the rate of orientation change about each axis of the sensor frame. The relative orientation at the time of interest is obtained by integrating the raw measurement data for all rotational axes. The Euler angles are updated based on the gyroscope readings as follows:

(17) $$\begin{array}{cc} \theta_{k} & =\theta_{k-1}+\omega y_{k}T_{s}\\ \phi_{k} & =\phi_{k-1}+\omega x_{k}T_{s}\\ \psi_{k} & =\psi_{k-1}+\omega z_{k}T_{s} \end{array}$$

where $\omega x_{k}$, $\omega y_{k}$, and $\omega z_{k}$ are the gyroscope readings of the angular rotation rate about the X-, Y-, and Z-axes, respectively, and $T_{s}$ is the sampling time. On the other hand, the quaternion parameter time update equations based on gyroscope readings are also formulated as follows. From the quaternion identity,

(18) $${\left(p⊗q\right)}^{*}=q^{*}⊗p^{*}$$

The time derivative of quaternion is

(19) $$\frac{d(q^{*}⊗q)}{dt}={\dot{q}}^{*}⊗q^{*}+q^{*}⊗\dot{q}=0$$

It follows that

(20) $$q^{*}⊗\dot{q}=-\left({\dot{q}}^{*}⊗q\right)=-{\left(q^{*}⊗\dot{q}\right)}^{*}$$

This means that $q^{*}⊗\dot{q}$ is a pure quaternion (i.e., it is equal to the negative of its conjugate; therefore, its real part is zero). Thus, we take a pure quaternion Ω and write:

(21) $$q^{*}⊗\dot{q}=\Omega =\left[\begin{array}{c} 0\\ \Omega \end{array}\right]$$

Left multiplication by **q** yields the differential equation

(22) $$\dot{q}=q⊗\Omega =\frac{1}{2}q⊗\omega$$

Converting Equation (22) to discrete time with sample time ΔT and taking the first order approximation of its Taylor series expansion yields Equation (23)

(23) $$q_{k}=\left(\begin{array}{c} I_{4x4}+\frac{1}{2}\Omega_{k}T_{s} \end{array}\right)q_{k-1}$$

where

$$\Omega_{k}=\frac{1}{2}\left[\begin{array}{cc} 0 & -\omega^{T}\\ \omega & -[\omega_{x}] \end{array}\right]=\frac{1}{2}\left[\begin{array}{cccc} 0 & -\omega_{x} & -\omega_{y} & -\omega_{z}\\ \omega_{x} & 0 & \omega_{z} & -\omega_{y}\\ \omega_{y} & -\omega_{z} & 0 & \omega_{x}\\ \omega_{z} & \omega_{y} & -\omega_{x} & 0 \end{array}\right],\left[\omega_{x}\right]=\left[\begin{array}{ccc} 0 & -\omega_{z} & \omega_{y}\\ \omega_{z} & 0 & -\omega_{x}\\ -\omega_{y} & \omega_{x} & 0 \end{array}\right]$$

and

$$\omega ={\left[\begin{array}{ccc} \omega_{x} & \omega_{y} & \omega_{z} \end{array}\right]}^{T}$$

## 3. Attitude and Heading Estimation with EKF-Based Sensor Fusion

As discussed in Section 2, Euler’s and quaternion parameters are used to express attitudes and heading. The Euler representation of angular rotation is quite intuitive to use but the quaternion representation is generally used in UAV applications. This is because 3D rotation expression with quaternion numbers does not cause gimbal lock problem [19], and conversely, the 3D rotation expression with Euler angles is susceptible to the gimbal lock problem. As a result, a quaternion-based approach for attitude and heading estimation was preferred for this study.

In Section 2, the direct computation of attitude and heading from the gyroscope, accelerometer, and magnetometer data is presented. However, the results that were based on gyroscope measurements accumulated errors over each calculation, leading to more severe drift over time. On the other hand, the values that were based on the accelerometer and magnetometer readings were also distorted by noise signals but were not affected by drift over time. Both of these methods have their drawbacks, but when used together, they can correct one another. Therefore, it is essential to predict the attitude and heading with the gyroscope in advance and to use the accelerometer and magnetometer as correctors to obtain reliable results using the EKF algorithm.

### 3.1. EKF Formulation

The EKF is an iterative prediction/correction approach for estimating the state of a discrete time process or measurement. Before moving on to the algorithm, it is important to select either the quaternion or Euler representation method. In this work, the quaternion-based approach is preferred because this approach does not introduce the gimbal lock problem. The EKF process relies on a state prediction model that mathematically defines how the state variable $x_{k}$ is related to the input variable and time, and an observation model that establishes a mathematical connection between the measured values $z_{k}$ and the predicted states $x_{k}$.

(24) $$\left\{\begin{array}{c} x_{k}=F_{k}x_{k-1}+\omega_{k}\\ z_{k}=H_{k}x_{k}+\nu_{k} \end{array}\right.,\;where\;\left\{\begin{array}{c} x_{k}\text{:}\;\mathrm{state}\;\mathrm{at}\;\mathrm{sampling}\;\mathrm{time}\;k\;\\ z_{k}\text{:}\;\mathrm{measurement}\;\mathrm{at}\;\mathrm{sampling}\;\mathrm{time}\;k\;(\;\mathrm{or}\;\mathrm{observation})\\ F_{k}\text{:}\;\mathrm{state}\;\mathrm{transition}\;\mathrm{model}\;\mathrm{applied}\;\mathrm{to}\;x_{k-1}\;\\ H_{k}\text{:}\;\mathrm{observation}\;\mathrm{model} \end{array}\right.$$

The EKF algorithm follows three major sequential steps: initialization, prediction and correction.

#### 3.1.1. Initialization

At this stage, the state values are set to their original orientations, which in most cases start at zero rotation about all axes, described with quaternions $q={\left[\begin{array}{cccc} 1 & 0 & 0 & 0 \end{array}\right]}^{T}$.

(25) $$\begin{array}{cc} \; & \hat{q_{0}}={\left[\begin{array}{cccc} 1 & 0 & 0 & 0 \end{array}\right]}^{T}\\ \; & P_{0}=E\left[\begin{array}{c} \left(q_{0}-\hat{q_{0}}\right){\left(q_{0}-\hat{q_{0}}\right)}^{T} \end{array}\right] \end{array}$$

The measurement noise covariance matrix (*R*) and the process noise covariance matrix (*Q*) can be time-varying or time-invariant. The heading estimation is highly sensitive to magnetic noise. Therefore, in this study, simulations and practical experiments were conducted to determine how to minimize magnetic disturbances in heading estimation by using a disturbance-dependent measurement covariance matrix; this topic is further discussed in Section 3.3. The initializations of *Q* and *R* are given in Equation (26)

(26) $$R=\sigma_{\nu }I_{mxm},\;Q=\sigma_{\omega }I_{nxn}$$

where *I*, *n*, and *m* are the identity matrix, number of states, and number of measurements, respectively. $\sigma_{\nu }$ and $\sigma_{\omega }$ are the variance of the measurement noise and the variance of the process noise, respectively.

#### 3.1.2. Prediction

A mathematical model of quaternion-based orientation estimation is presented in Section 2.2. Based on previous sensor states, the sample period, and current gyroscope measurements, the prediction equation helps to calculate the sensor’s attitude and heading. The outcome of this stage is then used in the EKF fusion process. The model given in Equation (23) is eventually reformulated in the standard state-space model form, as shown in Equation (27).

(27) $$q_{k}^{-}=F_{k}q_{k-1},\;where,\;F_{k}=\left[\begin{array}{cccc} 1 & -\omega_{x}\frac{T_{s}}{2} & -\omega_{y}\frac{T_{s}}{2} & -\omega_{z}\frac{T_{s}}{2}\\ \omega_{x}\frac{T_{s}}{2} & 1 & \omega_{z}\frac{T_{s}}{2} & -\omega_{y}\frac{T_{s}}{2}\\ \omega_{y}\frac{T_{s}}{2} & -\omega_{z}\frac{T_{s}}{2} & 1 & \omega_{x}\frac{T_{s}}{2}\\ \omega_{z}\frac{T_{s}}{2} & \omega_{y}\frac{T_{s}}{2} & -\omega_{x}\frac{T_{s}}{2} & 1 \end{array}\right]$$

Additionally, the prediction error covariance is

(28) $$P_{k}^{-}=F_{k}P_{k-1}F_{k}^{T}+Q$$

The observation model, as shown in Equations (9) and (11) for the accelerometer and magnetometer, respectively, can easily be defined in a combination form.

(29) $$z_{k}=\left[\begin{array}{c} a_{x}\\ a_{y}\\ a_{z}\\ m_{x}\\ m_{y}\\ m_{z} \end{array}\right]=\left[\begin{array}{c} 2(q_{1}q_{3}-q_{0}q_{2})\\ 2(q_{2}q_{3}+q_{0}q_{1})\\ q_{0}^{2}-q_{1}^{2}-q_{2}^{2}+q_{3}^{2}\\ \left(q_{0}^{2}+q_{1}^{2}-q_{2}^{2}-q_{3}^{2}\right)H_{x}+2\left(q_{1}q_{2}+q_{0}q_{3}\right)H_{y}+2\left(q_{1}q_{3}-q_{0}q_{2}\right)H_{z}\\ 2\left(q_{1}q_{2}-q_{0}q_{3}\right)H_{x}+\left(q_{0}^{2}-q_{1}^{2}+q_{2}^{2}-q_{3}^{2}\right)H_{y}+2\left(q_{2}q_{3}+q_{0}q_{1}\right)H_{z}\\ 2\left(q_{1}q_{3}+q_{0}q_{2}\right)H_{x}+2\left(q_{2}q_{3}-q_{0}q_{1}\right)H_{y}+\left(q_{0}^{2}-q_{1}^{2}-q_{2}^{2}+q_{3}^{2}\right)H_{z} \end{array}\right]=h\left(q_{k}^{-}\right)$$

Then,

(30) $$\begin{array}{cc} H_{k} & =\frac{\partial h}{\partial q}{|}_{q=q_{k-1}}\\ \; & =\left[\begin{array}{ccc} -2q_{2} & 2q_{3} & -2q_{0}\\ 2q_{1} & 2q_{0} & 2q_{3}\\ 2q_{0} & -2q_{1} & -2q_{2}\\ 2q_{0}H_{x}+2q_{3}H_{y}-2q_{2}H_{z} & 2q_{1}H_{x}+2q_{2}H_{y}+2q_{3}H_{z} & -2q_{2}H_{x}+2q_{1}H_{y}-2q_{0}H_{z}\\ -2q_{3}H_{x}+2q_{0}H_{y}+2q_{1}H_{z} & 2q_{2}H_{x}-2q_{1}H_{y}+2q_{0}H_{z} & 2q_{1}H_{x}+2q_{2}H_{y}+2q_{3}H_{z}\\ 2q_{2}H_{x}-2q_{1}H_{y}+2q_{0}H_{z} & 2q_{3}H_{x}-2q_{0}H_{y}-2q_{1}H_{z} & 2q_{0}H_{x}+2q_{3}H_{y}-2q_{2}H_{z} \end{array}\right.\\ \; & \;\;\;\;\;\;\;\;\;\;\;\;\;\left.\begin{array}{c} 2q_{1}\\ 2q_{2}\\ 2q_{3}\\ -2q_{3}H_{x}+2q_{0}H_{y}+2q_{1}H_{z}\\ -2q_{0}H_{x}-2q_{3}H_{y}+2q_{2}H_{z}\\ 2q_{1}H_{x}+2q_{2}H_{y}+2q_{3}H_{z} \end{array}\right] \end{array}$$

#### 3.1.3. Correction

This is the last step of every single iteration of the EKF. The states and covariance matrix were corrected using the Kalman gain.

(31) $$\begin{array}{cc} \; & K=P_{k}^{-}H_{k}^{T}{\left(H_{k}P_{k}^{-}H_{k}^{T}+R\right)}^{-1}\\ \; & q_{k}^{+}=q_{k}^{-}+K\left(z_{k}-h\left(q_{k}^{-}\right)\right)\\ \; & P_{k}^{+}=\left(I-KH_{k}\right)P_{k}^{-} \end{array}$$

### 3.2. Double Quaternion Approach

In this study, a double quaternion configuration was proposed to represent the attitude and heading separately. A single quaternion consists of four parameters ($q={\left[q_{0}\;q_{1}\;q_{2}\;q_{3}\right]}^{T}$). However, the proposed double quaternion is, set to have eight parameters that are represented as ($q={\left[\begin{array}{ccccccc} q_{0} & q_{1} & q_{2}\;q_{3} & q_{w} & q_{x} & q_{y} & q_{z} \end{array}\right]}^{T}$); the first four parameters are assigned to represent attitude, while the last four are assigned to represent heading information.

#### 3.2.1. Double Quaternion-Based EKF (DQEKF) Formulation

Many studies dealing with quaternion-based attitude and heading estimation use a single quaternion to denote both attitude and heading during the EKF updating process [1,4,20]. The update process for a single quaternion value, which can be seen in Equations (27) and (29), depends on three sensor values: gyroscope, accelerometer, and magnetometer. The information from the gyroscope is essentially used to predict attitude and heading, whereas the accelerometer and magnetometer are used to correct the prediction. However, magnetometer readings are more susceptible to environmental disturbances and can induce attitude errors if used for correction. Therefore, this study proposes an independent updating mechanism for attitude and heading to overcome the aforementioned problem. The EKF is formulated such that the attitude solely depends on accelerometer readings, whereas the heading relies on both the accelerometer as a tilt compensator and the magnetometer as a prediction error corrector.

##### Prediction

Owing to the independent updates of attitude and heading, the number of states is doubled.

(32) $$q^{d}={\left[\underset{q_{att}}{\underset{︸}{\begin{array}{cccc} q_{0} & q_{1} & q_{2} & q_{3} \end{array}}}\;\underset{q_{hdg}}{\underset{︸}{\begin{array}{cccc} q_{w} & q_{x} & q_{y} & q_{z} \end{array}}}\right]}^{T}$$

where $q^{d}$, $q_{att}$, and $q_{hdg}$ represent the double quaternion, quaternion corresponding to attitude, and quaternion corresponding to heading, respectively. Following the increase in the number of states, the prediction and observation models also change. The state transition matrix in Equation (27) is modified to

(33) $$F_{k}=\left[\begin{array}{cccccccc} 1 & -\omega_{x}\frac{T_{s}}{2} & -\omega_{y}\frac{T_{s}}{2} & -\omega_{z}\frac{T_{s}}{2} & 0 & 0 & 0 & 0\\ \omega_{x}\frac{T_{s}}{2} & 1 & w_{2}\frac{T_{s}}{2} & -\omega_{y}\frac{T_{s}}{2} & 0 & 0 & 0 & 0\\ \omega_{y}\frac{T_{s}}{2} & -\omega_{z}\frac{T_{s}}{2} & 1 & \omega_{x}\frac{T_{s}}{2} & 0 & 0 & 0 & 0\\ \omega_{z}\frac{T_{s}}{2} & \omega_{y}\frac{T_{s}}{2} & -\omega_{x}\frac{T_{s}}{2} & 1 & 0 & 0 & 0 & 0\\ 0 & 0 & 0 & 0 & 1 & -\omega_{x}\frac{T_{s}}{2} & -\omega_{y}\frac{T_{s}}{2} & -\omega_{z}\frac{T_{s}}{2}\\ 0 & 0 & 0 & 0 & \omega_{x}\frac{T_{s}}{2} & 1 & w_{2}\frac{T_{s}}{2} & -\omega_{y}\frac{T_{s}}{2}\\ 0 & 0 & 0 & 0 & \omega_{y}\frac{T_{s}}{2} & -\omega_{z}\frac{T_{s}}{2} & 1 & \omega_{x}\frac{T_{s}}{2}\\ 0 & 0 & 0 & 0 & \omega_{z}\frac{T_{s}}{2} & \omega_{y}\frac{T_{s}}{2} & -\omega_{x}\frac{T_{s}}{2} & 1 \end{array}\right]$$

and the observation model in Equation (30) expands to Equation (34)

(34) $$H_{k}=\left[\begin{array}{cc} H_{a}^{1} & 0_{3x4}\\ 0_{3x4} & H_{a}^{2}\\ 0_{3x4} & H_{m} \end{array}\right]$$

where

$$H_{a}^{1}=\left[\begin{array}{cccc} -2q_{2} & 2q_{3} & -2q_{0} & 2q_{1}\\ 2q_{1} & 2q_{0} & 2q_{3} & 2q_{2}\\ 2q_{0} & -2q_{1} & -2q_{2} & 2q_{3} \end{array}\right],\;H_{a}^{2}=\left[\begin{array}{cccc} -2q_{y} & 2q_{z} & -2q_{w} & 2q_{x}\\ 2q_{x} & 2q_{w} & 2q_{z} & 2q_{y}\\ 2q_{w} & -2q_{x} & -2q_{y} & 2q_{z} \end{array}\right]and$$

$$\begin{array}{cc} H_{m} & =\left[\begin{array}{ccc} 2q_{w}H_{x}+2q_{z}H_{y}-2q_{y}H_{z} & 2q_{x}H_{x}+2q_{y}H_{y}+2q_{z}H_{z} & -2q_{y}H_{x}+2q_{x}H_{y}-2q_{w}H_{z}\\ -2q_{z}H_{x}+2q_{w}H_{y}+2q_{x}H_{z} & 2q_{y}H_{x}-2q_{x}H_{y}+2q_{w}H_{z} & 2q_{x}H_{x}+2q_{y}H_{y}+2q_{z}H_{z}\\ 2q_{y}H_{x}-2q_{x}H_{y}+2q_{w}H_{z} & 2q_{z}H_{x}-2q_{w}H_{y}-2q_{x}H_{z} & 2q_{w}H_{x}+2q_{z}H_{y}-2q_{y}H_{z} \end{array}\right.\\ \; & \;\;\;\;\;\;\;\;\;\;\;\;\;\left.\begin{array}{c} -2q_{z}H_{x}+2q_{w}H_{y}+2q_{x}H_{z}\\ -2q_{w}H_{x}-2q_{z}H_{y}+2q_{y}H_{z}\\ 2q_{x}H_{x}+2q_{y}H_{y}+2q_{z}H_{z} \end{array}\right] \end{array}$$

The measurement and processing of noise covariance matrices obviously changes according to the current number of measurement and state variables.

#### 3.2.2. Observability Analysis

An observability analysis approach was applied to test the impact of faulty readings of the magnetometer sensor on attitude estimation. The observability of the quaternion parameters by the magnetometer sensor was analyzed by considering the observation model and state model given in Equations (14) and (27), respectively. For a single quaternion representation, when all states (quaternion parameters) are observable, interference with the magnetometer readings affects the attitude and heading output. However, in the case of a double quaternion, when the first four states (attitude quaternion parameters) are unobservable by the magnetometer sensor, no interference in the magnetometer measurement can affect the attitude outputs. Equations (37) and (38) show the observability analysis for both scenarios. The observability of nonlinear system is commonly addressed by a Lie derivative approach [21,22]. For a generic nonlinear system represented in state space as

(35) $$\Sigma :\left\{\begin{array}{c} \dot{x}\left(t\right)=f\left(x\left(t\right),u\left(t\right)\right)=f^{0}\left(x\left(t\right)\right)+\sum_{i=1}^{l}f^{i}\left(x\left(t\right)\right)u_{i}\left(t\right)\\ y=h(x), \end{array}\right.$$

where $x={\left[x_{1},x_{2},\cdots ,x_{n}\right]}^{T}$, $u={\left[u_{1},u_{2},\cdots ,u_{l}\right]}^{T}$ and $y={\left[y_{1},y_{2},\cdots ,y_{m}\right]}^{T}$ represent state, input and output measurements, respectively. The Lie derivative is

(36) $$L_{f}^{i}\left(h\right)=\left\{\begin{array}{c} h;\;\;\;\;\mathrm{for}\;i\;=\;0\\ \frac{\partial }{\partial x}\left[L_{f}^{i-1}\left(h\right)\right]f;\;\;\mathrm{for}\;i=1,2,3,\ldots ,\;n \end{array}\right.$$

For a single quaternion representation case, by using the system state model in Equation (27) and the magnetometer sensor state observation model in Equation (14), the observability matrix can be derived, as presented in Equation (37). Detailed derivations for Equations (37) and (38) are provided in Appendix A.

(37) $$O_{1}={\left[\begin{array}{cccc} \frac{\partial }{\partial q}\left(\begin{array}{c} L_{f}^{0}\left(h\right) \end{array}\right) & \frac{\partial }{\partial q}\left(\begin{array}{c} L_{f}^{1}\left(h\right) \end{array}\right) & \frac{\partial }{\partial q}\left(\begin{array}{c} L_{f}^{2}\left(h\right) \end{array}\right) & \frac{\partial }{\partial q}\left(\begin{array}{c} L_{f}^{3}\left(h\right) \end{array}\right) \end{array}\right]}^{T}$$

Similarly, for the double quaternion representation case, based on the state model from Equation (33) and the observation model from Equation (14), the observability matrix is formulated as Equation (38).

(38) $$\begin{array}{cc} O_{2} & =\left[\begin{array}{cccc} \frac{\partial }{\partial q^{d}}\left(\begin{array}{c} L_{f}^{0}\left(h\right) \end{array}\right) & \frac{\partial }{\partial q^{d}}\left(\begin{array}{c} L_{f}^{1}\left(h\right) \end{array}\right) & \frac{\partial }{\partial q^{d}}\left(\begin{array}{c} L_{f}^{2}\left(h\right) \end{array}\right) & \frac{\partial }{\partial q^{d}}\left(\begin{array}{c} L_{f}^{3}\left(h\right) \end{array}\right) \end{array}\right.\\ \; & \;{\left.\begin{array}{cccc} \frac{\partial }{\partial q^{d}}\left(\begin{array}{c} L_{f}^{4}\left(h\right) \end{array}\right) & \frac{\partial }{\partial q^{d}}\left(\begin{array}{c} L_{f}^{5}\left(h\right) \end{array}\right) & \frac{\partial }{\partial q^{d}}\left(\begin{array}{c} L_{f}^{6}\left(h\right) \end{array}\right) & \frac{\partial }{\partial q^{d}}\left(\begin{array}{c} L_{f}^{7}\left(h\right) \end{array}\right) \end{array}\right]}^{T} \end{array}$$

The observability matrix $O_{1}$ is full rank, implying that all single quaternion parameters are observable so that attitude values are susceptible to magnetic interference. The rank of $O_{2}$ is four, which is less than the number of states by half. Therefore, four of the quaternion parameters out of eight are unobservable, which is a good indication that magnetic interference-immune quaternion parameters exist.

### 3.3. Magnetic Disturbance Tolerant Heading Estimation Mechanism

A magnetic sensor measures the strength of Earth’s magnetic field in the sensor frame. Earth’s magnetic field varies across geographic locations. According to the literature, the approximate magnetic field of the Earth can be referenced from the world magnetic model [23]. Therefore, identifying the magnitude of the magnetic disturbance at a specific location qualifies mathematically. In this subsection, a mathematical methods for identifying magnetic disturbances and for taking appropriate action will be discussed.

Assume that $H_{k}={\left[H_{x}\;H_{y}\;H_{z}\right]}^{T}$ represents the Earth’s magnetic field at a given time and at geographic location $P={\left[lat\;lon\;alt\right]}^{T}$. Then, the magnetic disturbance distribution at a given location is estimated as

(39) $$d_{m}=\sum_{k-n}^{k}\left(\right|H_{k}\left|-\right|B_{k}{\left|\right)}^{2}$$

where |H|, |B|, and n represent the magnitude of the Earth’s magnetic field at a location, the magnitude of the magnetic field measured by the magnetometer, and the number of samples considered, respectively. The number of samples was decided based on how fast the system should respond and how long the disturbance should last, as considered by the system.

**Figure 2:**
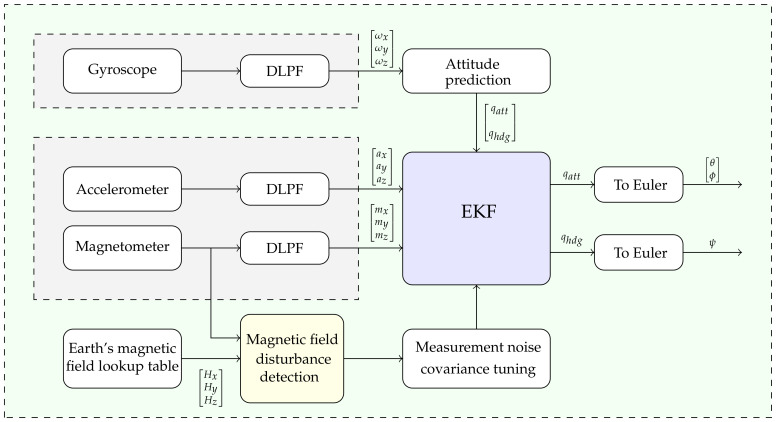
Attitude and heading estimation algorithm block diagram.

Based on the error calculated in Equation (39), a rule was created to determine the error condition for switching to different values of the measurement error covariance. The covariance value of the measurement error indicates the degree to which the system relies on the gyroscope prediction or magnetometer data to estimate the heading [24]. If a severe magnetic disturbance is detected, a big measurement error covariance value is selected to prevent the effect of erroneous sensor data on the estimate. The magnetic field disturbance detector compares Earth’s magnetic field measured by a magnetometer sensor with Earth’s magnetic field referred from a lookup table that contains Earth’s magnetic field based on location and projected onto the sensor frame. The proposed algorithm for magnetic disturbance detection and rejection, as well as the decoupling of attitude calculation from heading, is shown in Figure 2.

## 4. Hardware Design

For functional verification of the proposed algorithm, an embedded inertial navigation system (INS) prototype was developed in this study. The prototype was equipped with an ICM-20948 9-axis MEMS TDK InvenSense $MotionTracking^{TM}$ device, and a Texas Instruments TMS320F28377S MCU for the proposed EKF algorithm computation and communication with peripheral devices.

Table 1 and Table 2 summarize the specifications of the ICM-20948 and TMS320F28377S MCUs, respectively.

**Figure 3:**
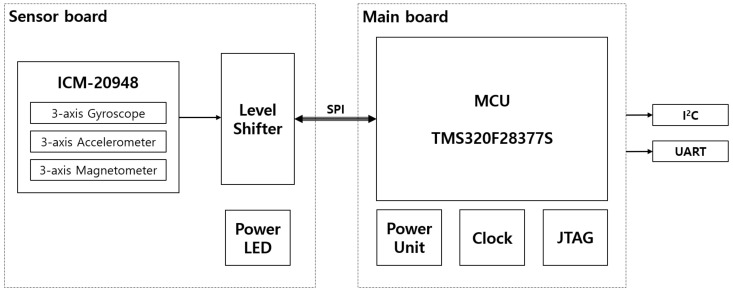
Block diagram of the INS prototype.

The PCB of the prototype included a sensor board with sensor chips and a main board with an MCU, a power source, and external interface connectors. Figure 3 shows the configuration of the INS prototype.

**Figure 4:**
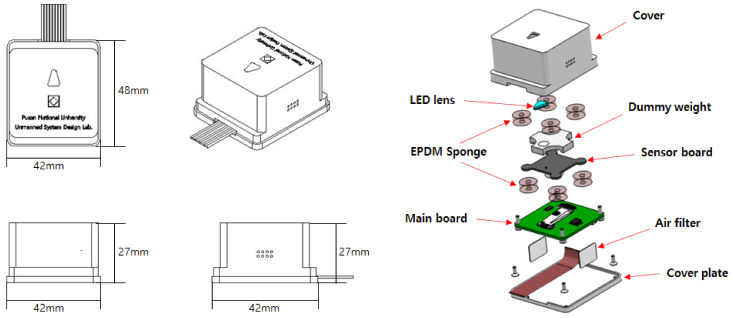
Configuration of the INS prototype.

**Figure 5:**
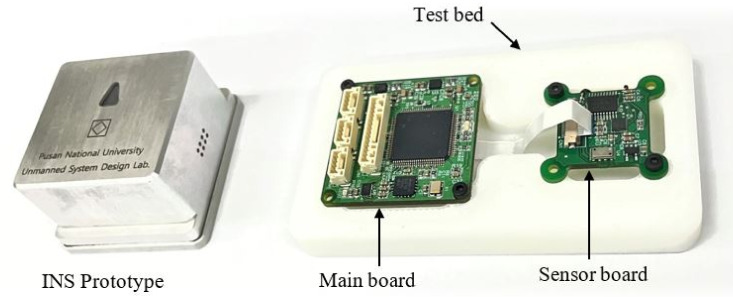
Picture of developed INS module.

The sensor board and the main board are connected by a flexible flat cable and are designed to be physically secured with double-sided tape made of a soft ethylene propylene diene monomer (EPDM) sponge material. This is to minimize the effect of vibration due to the actuator operation of the INS mounted targets, such as drones and ground robots. Figure 4 shows the designed assembly configuration of the inner PCB parts and the outer cover of the prototype, and Figure 5 shows the actual sensor board and main board.

## 5. Computer Simulation and Experimentation

### 5.1. Sensor Data Generation

Simulated IMU sensor data were generated to compare the performance of complementary, gradient descent proposed by Madgwick, EKF, and the proposed double quaternion-based EKF algorithms in estimating attitude and heading, and in eliminating environmental magnetic disturbance. MATLAB software was used to generate the IMU sensor data using the parameters listed in Table 3.

**Figure 6:**
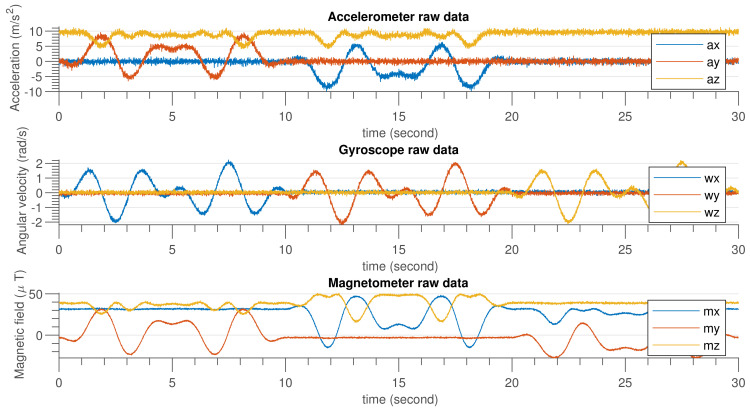
Accelerometer, gyroscope and magnetometer simulated data.

**Figure 7:**
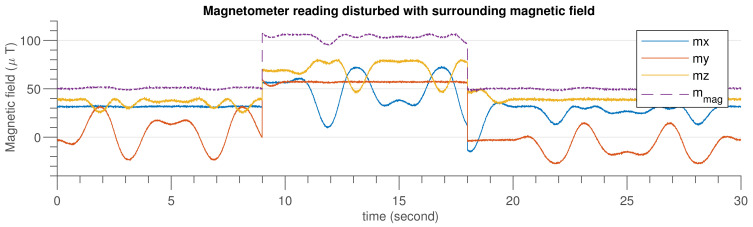
Magnetometer simulated data after injecting external disturbance.

Figure 6 shows the IMU sensor data generated using MATLAB code by considering the specification given in Table 3. In Figure 7, an emulated external magnetic interference signal that was applied at the ninth second and lasted until the 18th second is shown.

### 5.2. Experimental Setup

**Figure 8:**
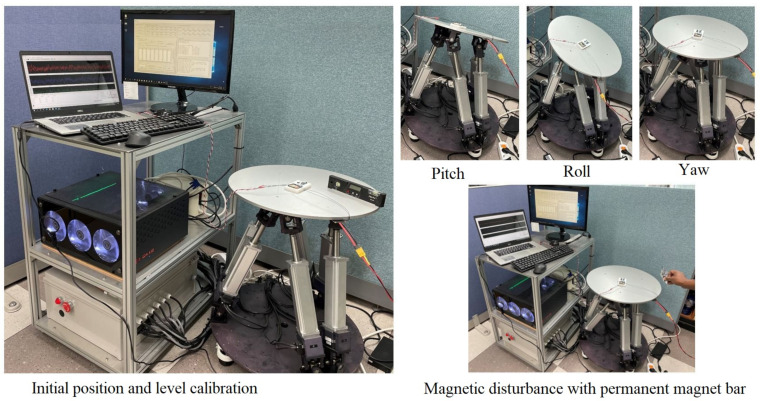
Experimental setup.

In order to carry out a validation experiment for the proposed algorithm, an experimental environment was set up as shown in Figure 8. A six D.O.F motion table, computer, spirit level, and permanent magnetic bar were used for the experiment. The usage of these tools is described briefly as follows.

- Motion table: This machine was used to rotate the sensor around the X-, Y-, and Z-axes to an orientation angle required by the computer software. The software was developed as real-time harware-in-the-loop simulation (HILS) testing tool for small UAVs [25]. The specifications of the motion table are given in Table 4;
- Computer: A command is sent to the motion table from the computer. The raw data measured by the sensors and the estimated orientation angles are also logged into a file on the computer;
- Permanent magnet: We used a permanent magnetic bar to create a magnetic disturbance in the environment.

In the experiment, the magnetometer was carefully calibrated, as the workplace area was filled with different tools that could contain metallic materials that would create magnetic interference and affect the output of the experiment. Then, the motion table was operated to rotate around the X-, Y-, and Z-axes in sequence for specific times, under conditions with no significant magnetic disturbance, and in a magnetically disturbed environment by placing a magnetic bar in close vicinity to the motion table. Experiments were also carried out when the motion table was not moving to compare performance under static and dynamic conditions. Simultaneously, all raw measurement data from the sensors and estimated rotation trajectories were recorded.

## 6. Results

### 6.1. Simulation Result

**Figure 9:**
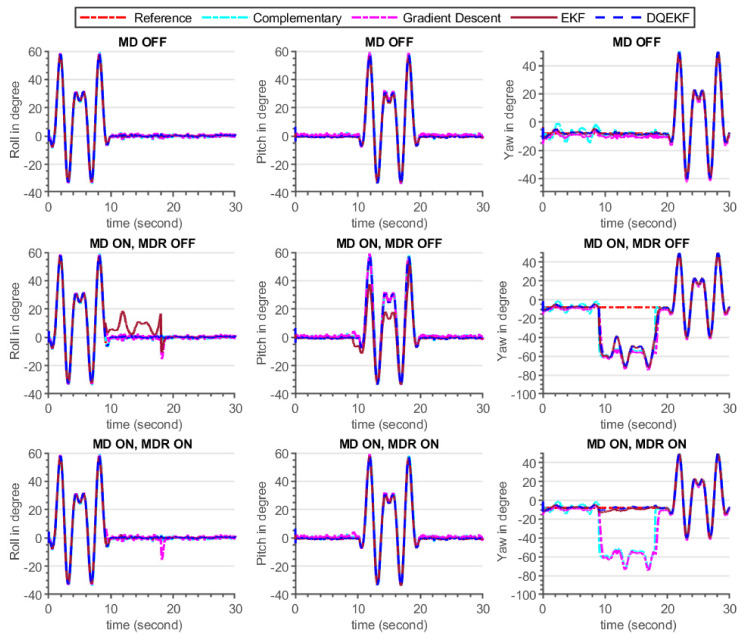
Attitude and heading estimation by complementary, gradient descent, EKF, and double quaternion-based EKF for three cases (Case 1: no magnetic disturbance (MD OFF), Case 2: with magnetic disturbance and no magnetic disturbance rejection (MD ON, MDR OFF), Case 3: with both magnetic disturbance and magnetic disturbance rejection enabled (MD ON, MDR ON)).

In this simulation experiment, comparisons of the complementary [26], gradient descent [27], EKF, and DQEKF algorithms were carried out. Two major concerns were examined while comparing the performances of the aforementioned algorithms. The first issue deals on the decoupling of attitude and heading estimation in order to eliminate the effect of magnetometer reading on attitude (roll and pitch) computation. Second, an environmental magnetic disturbance rejection system was considered for comparison. The performance of each algorithm in response to the injected magnetic disturbance shown in Figure 9 and Table 5.

### 6.2. Experimental Result

**Figure 10:**
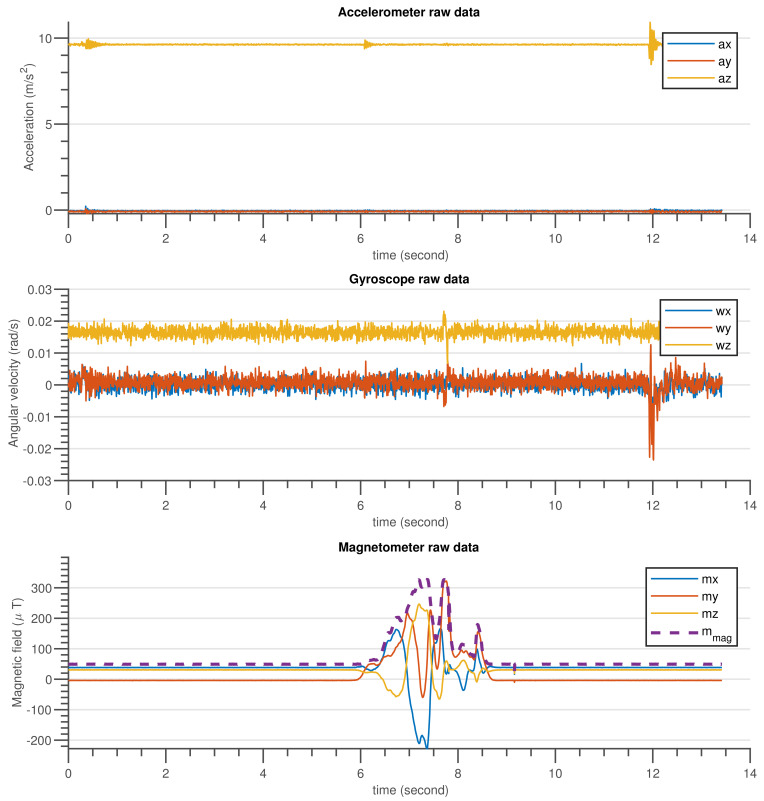
The gyroscope, accelerometer and magnetometer measurement data when INS sensor was stationary, and disturbed by temporary magnetic noise signal for a short time.

**Figure 11:**
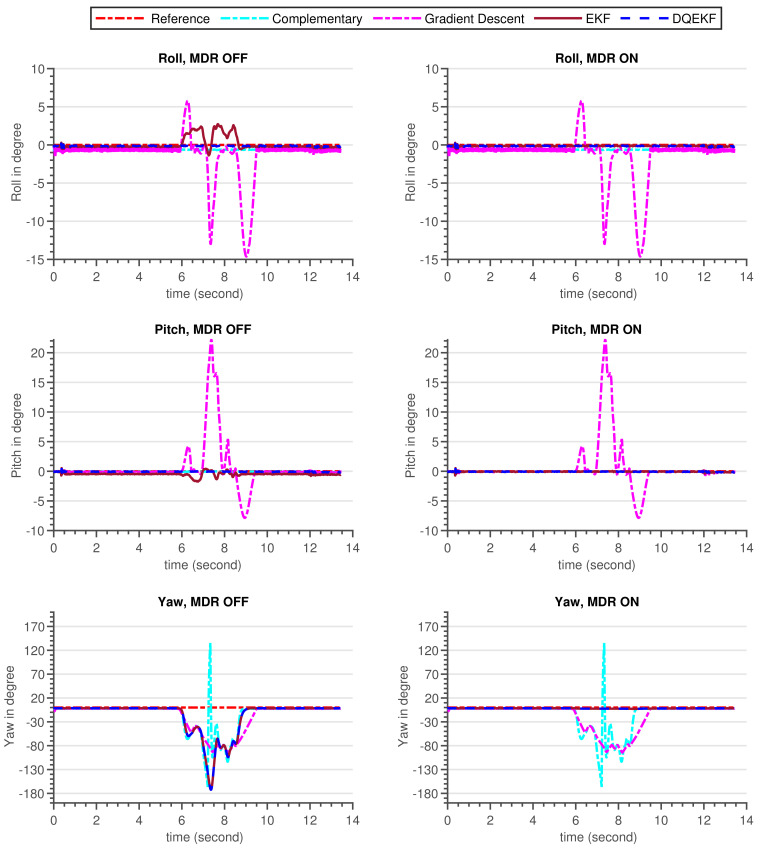
Euler angle estimation output by complementary, gradient descent, EKF and DQEKF algorithms when temporary magnetic field was introduced in the environment for a short period of time and the INS sensor was stationary.

**Figure 12:**
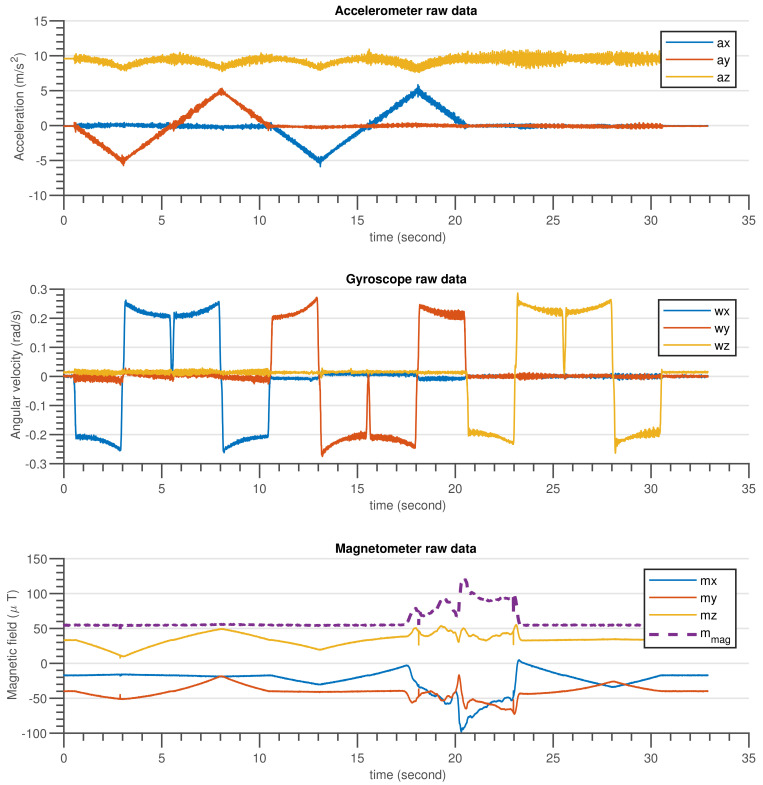
The gyroscope, accelerometer and magnetometer measurement data when the INS sensor was rotated by the motion table and disturbed by temporary magnetic noise signal for a short time.

**Figure 13:**
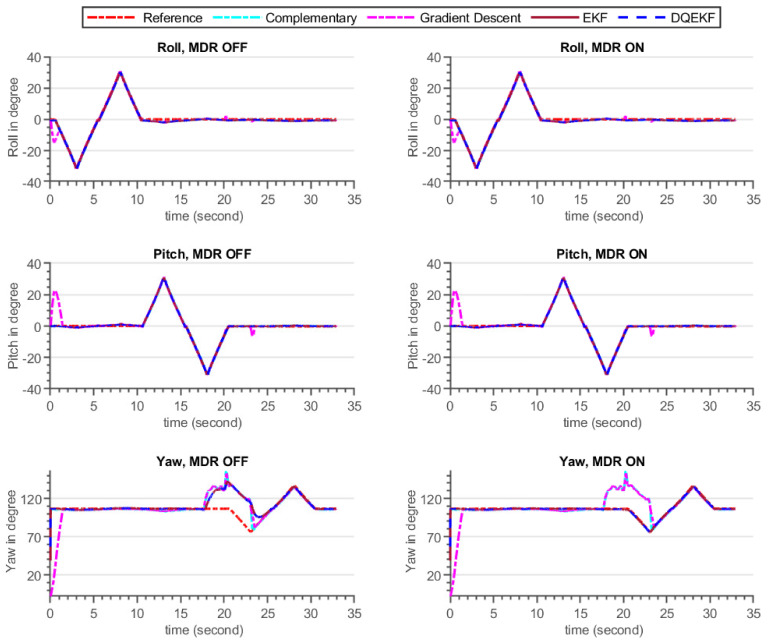
Euler angle estimation output by complementary, gradient descent, EKF and DQEKF algorithms when temporary magnetic field was introduced in the environment for a short period of time, and the INS sensor was rotating by the motion table.

The validation procedure was carried out using the experimental setup specified in Section 5.2. A high-precision motion table was utilized to rotate the developed sensor around the inertial axes and serve as a ground truth reference. The performance of the built AHRS with the proposed algorithm and other commonly used algorithms was carefully examined in this experiment, for avoiding the influence of environmental magnetic disturbance on attitude and heading estimation. A permanent magnet was utilized to create an artificial magnetic disturbance in the surroundings. Figure 10 and Figure 11 show the raw sensors measurement data and the Euler angles estimated by the respective algorithms: complementary, gradient descent, EKF, and DQEK, respectively, while the INS sensor was stationary and magnetic disturbance was injected for small period of times. Furthermore, the same experimental scenario followed while the INS sensor was rotating by the motion table and the recorded results depicted in Figure 12 and Figure 13. Table 6 presents the empirical data analysis of the algorithms.

## 7. Discussion

This study covered all of the processes needed to create a fully working AHRS sensor, including algorithm development and simulation validation, as well as sensor hardware design, manufacturing, and experimental verification. Furthermore, the common algorithms complementary, gradient descent, and EKF were examined with the identical magnetic disturbance conditions as the double quaternion-based EKF to confirm the required performance by the developed algorithm and hardware.

### 7.1. Simulation Results Discussion

As seen in the simulation results demonstrated in Figure 9 and Table 5, complementary and DQEKF outperformed the other algorithms in terms of excluding the effect of magnetometer measurement on attitude estimation during magnetic disturbance condition, whereas gradient descent and EKF attitude estimation were directly affected by magnetometer noise because the attitude computation was not independent of the magnetometer readings. According to the analysis of observability of attitude by the magnetometer sensor discussed in Section 3.2.2, when employing the single quaternion-based EKF method, all four quaternion parameters were completely observable. This means that any changes in the magnetometer sensor data could have an effect on the attitude values. Therefore, the low performance of EKF and gradient descent approaches, during magnetic disturbance, was attributed to the coupling of magnetometer data and attitude in their mathematical models. Though complementary filter attitude estimation was unaffected by magnetic noise, its accuracy was lower than that of DQEKF since DQEKF was based on an efficient and advanced EKF algorithm.

Regarding heading estimation, DQEKF performed better than EKF in rejecting the magnetic disturbance, even if the same magnetic disturbance rejection algorithm was used in both cases. This is because the error in attitude due to disturbance also affects the heading. As the magnetic disturbance algorithm developed in this study could not be directly applied to the complementary and gradient descent algorithms, only the EKF and DQEKF comparisons were performed. Overall, it can be concluded that the EKF and DQEKF algorithms surpass the complementary and gradient descent algorithms in terms of the accuracy and reliability of the attitude and heading estimation. DQEKF is better than EKF in preventing the effect of magnetometer noise in both attitude and heading estimation.

### 7.2. Experimental Results Discussion

The experiments were carried out for static and dynamic statuses of the INS sensor. The results of the two experimental scenarios were consistent with one another and also with the simulation results discussed in Section 7.1. When the INS sensor is in a stationary state, all Euler angle values should remain in their initial states if the gyroscope biases are completely removed by calibration. In other words, the Euler angles will drift over time if the gyroscope sensor biases are not zero, particularly if the applied algorithm places more reliance on the gyroscope data. In the dynamic condition, the same issue arises. Nevertheless, corrective action based on accelerometer measurements can reduce drift in attitude angle (roll and pitch) estimation over time. Similarly, the heading drift is expected to be compensated using magnetometer sensor readings but the magnetometer itself is vulnerable to magnetic noise from the environment. As a consequence, reducing heading estimation inaccuracy is a tradeoff. Therefore, the solution strategy should be focused on determining when gyroscope or magnetometer reading data are more relevant.

The divergence of the heading (yaw) value from the reference value in Figure 11 and Figure 13 after the introduction of magnetic flux demonstrated that everything that happens to the magnetometer directly affects the heading estimated output, independent of the algorithms utilized, which included complementary, gradient descent, EKF, and DQEK. When the magnetic disturbance rejection (MDR) technique is applied with the EKF and DQEK algorithms, the degree to which the heading value is affected by the surrounding magnetic flux considerably reduced for both static and dynamic conditions. In terms of avoiding the environmental magnetic flux on heading estimated output, DQEKF surpasses EKF. The roll and pitch estimates of the gradient descent and EKF algorithms, however, are impacted by magnetic disturbance. The complementary and DQEKF algorithms proved to be unaffected by magnetic disturbances in the environment for attitude estimation. Owing to the overall accuracy, reliability, and simplicity of applying the magnetic disturbance rejection method, DQEKF was the best among the algorithms considered in this experiment. Generally, the severity of the magnetic flux effect on the roll and pitch depends on the accuracy of the gyroscope. If the gyroscope sensor used is of poor quality or not properly calibrated, magnetic disturbance will negatively affect the attitude value in addition to heading, and DQEKF should be used to solve the problem.

## 8. Conclusions

In this work, two complementary methods are presented for rejecting the effect of magnetic disturbance in determining a reliable 3-D orientation of a moving object such as a UAV. The first method addressed the issue of attitude estimation error due to the magnetic noise by using a double quaternion representation of an object orientation in the EKF algorithm process. With this method, the attitude and heading are set to update with independent quaternion parameters, where the quaternion parameters correspond to the attitude calculation decoupled from magnetometer readings. In the second method, the inaccuracy of the heading under magnetic interference is reduced by an online adjustment of the measurement error covariance matrix. The experimental tests proved that the deviation of attitude and heading values from the ground truth better reduced with the proposed methods than commonly used algorithms: complementary, gradient descent, and the conventional use of EKF, during the period of magnetic disturbance. Thus, in an environment where magnetic interference has a significant impact on the performance of attitude and heading sensors, the suggested approaches are solutions.

## Figures and Tables

**Table 1:** Specification of the ICM-20948.

Parameter	Gyroscope	Accelerometer	Magnetometer
Range	±2000 dps	±16 g	±4900 μT
Output Data Rate	1.125 kHz	1.125 kHz	100 Hz
Spectral noise density	0.015 dps/$\sqrt{H}z$	230 μg/$\sqrt{H}z$	
Interface	$I^{2}C$: 400 kHz, SPI: 7 MHz		
ADC word length	16 bits		

**Table 2:** Specification of the TMS320F28377S CPU.

Parameter	Value	Parameter	Value
Total Processing (MIPS)	400 MHz	ADC resolution	16-bit, 12-bit
Communication peripherals	2-CAN, 4-UART, 3-high speed SPI (up to 50 MHz), $I^{2}C$	Flash memory	1024 KB
		Mechanical dimension	256 mm${}^{2}$ 16 × 16

**Table 3:** Specification of simulated sensors.

	Bias	Noise Density
Gyroscope	[0.0428 −0.0327 0.0209] rad/s	[0.0100 0.0100 0.0100] rad/s/$\sqrt{Hz}$
Accelerometer	[−0.0599 −0.0042 −0.1780] m/$s^{2}$	[0.0730 0.0730 0.0730] m/s${}^{2}/\sqrt{H}z$
Magnetometer	[0.1000 0.1000 0.1000] μT	[0.0600 0.0600 0.0900] μT/$\sqrt{H}z$

**Table 4:** Specification of motion table.

Specification	Excursion	Vel.	Acc.
Roll	±30°	45°/s	1520°/s${}^{2}$
Pitch	±30°	45°/s	1520°/s${}^{2}$
Yaw	±60°	45°/s	5146°/s${}^{2}$

**Table 5:** RMS error of complementary, gradient descent, EKF, and double quaternion-based EKF algorithms comparison for attitude and heading estimation. MD and MDR stand for magnetic disturbance and magnetic disturbance rejection, respectively.

Algorithm	Euler Angles	RMS of Euler Angles Estimation Error in Degree for Three Different Magnetic Disturbance Cases		
		MD OFF	MD ON, MDR OFF	MD ON, MDR ON
Complementary (α=0.9)	Roll	1.4085	1.4085	
	Pitch	1.3413	1.3413	
	Yaw	1.8295	27.9305	
Gradient descent (β=0.9)	Roll	0.6410	2.4940	
	Pitch	0.5521	0.9618	
	Yaw	1.8648	28.0109	
EKF	Roll	0.5796	4.9787	0.6267
	Pitch	0.8518	5.5300	0.7289
	Yaw	0.9646	26.6888	1.6488
DQEKF (Proposed)	Roll	0.5933	0.5933	0.5933
	Pitch	0.6579	0.6579	0.6579
	Yaw	1.0279	26.4927	1.2574

**Table 6:** RMS error of comparison of complementary, gradient descent, EKF and double quaternion-based EKF algorithms for attitude and heading estimation. MD and MDR stand for magnetic disturbance and magnetic disturbance rejection, respectively.

Algorithm	Euler Angles Error RMS (degree)	Attitude and Heading Estimation When the Magnetic Disturbance Rejection (MDR) was ON or OFF			
		Static Condition		Dynamic Condition	
		MDR OFF	MDR ON	MDR OFF	MDR ON
Complementary (α=0.9)	Roll	0.6446		0.8521	
	Pitch	0.0765		0.4586	
	Yaw	34.4490		12.9683	
Gradient Descent (β=0.9)	Roll	3.2341		1.9019	
	Pitch	4.0225		3.2809	
	Yaw	33.2160		19.5133	
EKF	Roll	0.8057	0.1525	0.8405	0.8347
	Pitch	0.5548	0.0878	0.3769	0.3757
	Yaw	38.6632	1.7721	12.3855	1.3562
DQEKF (Proposed)	Roll	0.1523	0.1523	0.8044	0.8044
	Pitch	0.0867	0.0867	0.3738	0.3738
	Yaw	38.6684	1.7415	12.3545	0.9892

## Data Availability

Not applicable.

## Abbreviations

The following abbreviations are used in this manuscript:

MEMS	MicroElectroMechanical Systems
EKF	Extended Kalman Filter
DQEKF	Double Quaternion-based EKF
LKF	Linear Kalman Filter
UKF	Unscented Kalman Filter
PF	Particle Filter
CF	Complementary Filter
IMU	Inertial Measurement Unit
INS	Inertial Navigation System
MCU	Microcontroller Unit
AHRS	Attitude Heading and Reference System
UAV	Unmanned Aerial Vehicle
3D	Three Dimensional
PI	Proportional Integral
NED	North, East and Down
DLPF	Digital Low Pass Filter
RMS	Root Mean Square

## Appendix A. Observability Matrix Derivation

Single quaternion prediction model:

(A1) $$\begin{array}{cc} \; & q_{k}=f\left(q,\omega \right)=F_{k}q_{k-1}=\left[\begin{array}{c} q_{0}-0.5*T_{s}\left(\omega_{x}q_{1}+\omega_{y}q_{2}+\omega_{z}q_{3}\right)\\ q_{1}+0.5*T_{s}\left(\omega_{x}q_{0}+\omega_{z}q_{2}-\omega_{y}q_{3}\right)\\ q_{2}+0.5*T_{s}\left(\omega_{y}q_{1}-\omega_{z}q_{2}+\omega_{x}q_{3}\right)\\ q_{3}+0.5*T_{s}\left(\omega_{z}q_{1}+\omega_{y}q_{2}-\omega_{x}q_{3}\right) \end{array}\right]\\ \; & F_{k}=\frac{1}{2}\left[\begin{array}{cccc} 2 & -\omega_{x}T_{s} & -\omega_{y}T_{s} & -\omega_{z}T_{s}\\ \omega_{x}T_{s} & 2 & w_{z}T_{s} & -\omega_{y}T_{s}\\ \omega_{y}T_{s} & -\omega_{z}T_{s} & 2 & \omega_{x}T_{s}\\ \omega_{z}T_{s} & \omega_{y}T_{s} & -\omega_{x}T_{s} & 2 \end{array}\right]\\ \; & q={\left[\begin{array}{cccc} q_{0} & q_{1} & q_{2} & q_{3} \end{array}\right]}^{T} \end{array}$$

where ω is the angular rotation rate measured by the gyroscope sensor, $T_{s}$ is the sample time, and k is the time stamp.

Magnetometer observation model:

(A2) $$z=h\left(q\right)=\left[\begin{array}{c} \left(q_{0}^{2}+q_{1}^{2}-q_{2}^{2}-q_{3}^{2}\right)H_{x}+2\left(q_{1}q_{2}+q_{0}q_{3}\right)H_{y}+2\left(q_{1}q_{3}-q_{0}q_{2}\right)H_{z}\\ 2\left(q_{1}q_{2}-q_{0}q_{3}\right)H_{x}+\left(q_{0}^{2}-q_{1}^{2}+q_{2}^{2}-q_{3}^{2}\right)H_{y}+2\left(q_{2}q_{3}+q_{0}q_{1}\right)H_{z}\\ 2\left(q_{1}q_{3}+q_{0}q_{2}\right)H_{x}+2\left(q_{2}q_{3}-q_{0}q_{1}\right)H_{y}+\left(q_{0}^{2}-q_{1}^{2}-q_{2}^{2}+q_{3}^{2}\right)H_{z} \end{array}\right]$$

Lie derivative for single quaternion state representation (i.e., *n* = 4):

(A3) $$L_{f}^{i}\left(h\right)=\left\{\begin{array}{c} h(q);\;\;\;\;\mathrm{for}\;i\;=\;0\\ \frac{\partial }{\partial q}\left(\begin{array}{c} L_{f}^{i-1}\left(h\right) \end{array}\right)q_{k};\;\;\mathrm{for}\;i\;=\;1,2,3 \end{array}\right.$$

Then,

(A4) $$\begin{array}{cc} L_{f}^{0}\left(h\right) & =h(q)\\ L_{f}^{1}\left(h\right) & =\frac{\partial }{\partial q}\left(\begin{array}{c} L_{f}^{0}\left(h\right) \end{array}\right)q_{k}\\ L_{f}^{2}\left(h\right) & =\frac{\partial }{\partial q}\left(\begin{array}{c} L_{f}^{1}\left(h\right) \end{array}\right)q_{k}\\ L_{f}^{3}\left(h\right) & =\frac{\partial }{\partial q}\left(\begin{array}{c} L_{f}^{2}\left(h\right) \end{array}\right)q_{k} \end{array}$$

(A5) $$\begin{array}{cc} O_{1} & ={\left[\begin{array}{cccc} \frac{\partial }{\partial q}\left(\begin{array}{c} L_{f}^{0}\left(h\right) \end{array}\right) & \frac{\partial }{\partial q}\left(\begin{array}{c} L_{f}^{1}\left(h\right) \end{array}\right) & \frac{\partial }{\partial q}\left(\begin{array}{c} L_{f}^{2}\left(h\right) \end{array}\right) & \frac{\partial }{\partial q}\left(\begin{array}{c} L_{f}^{3}\left(h\right) \end{array}\right) \end{array}\right]}^{T}\\ R_{1} & =rank(O_{1}) \end{array}$$

$O_{1}$ is a 12 × 4 matrix. The rank of $O_{1}$ should be four for all parameters of a single quaternion (i.e., $q_{0}$, $q_{1}$, $q_{2}$ and $q_{3}$) to be observable by the magnetometer sensor.

Double quaternion prediction model:

(A6) $$\begin{array}{cc} \; & q_{k}^{d}=f\left(q^{d},\omega \right)=A_{k}q_{k-1}^{d}=\left[\begin{array}{c} q_{0}-0.5*T_{s}\left(\omega_{x}q_{1}+\omega_{y}q_{2}+\omega_{z}q_{3}\right)\\ q_{1}+0.5*T_{s}\left(\omega_{x}q_{0}+\omega_{z}q_{2}-\omega_{y}q_{3}\right)\\ q_{2}+0.5*T_{s}\left(\omega_{y}q_{0}-\omega_{z}q_{1}+\omega_{x}q_{3}\right)\\ q_{3}+0.5*T_{s}\left(\omega_{z}q_{0}+\omega_{y}q_{1}-\omega_{x}q_{2}\right)\\ q_{w}-0.5*T_{s}\left(\omega_{x}q_{x}+\omega_{y}q_{y}+\omega_{z}q_{z}\right)\\ q_{x}+0.5*T_{s}\left(\omega_{x}q_{w}+\omega_{z}q_{y}-\omega_{y}q_{z}\right)\\ q_{y}+0.5*T_{s}\left(\omega_{y}q_{w}-\omega_{z}q_{x}+\omega_{x}q_{z}\right)\\ q_{z}+0.5*T_{s}\left(\omega_{z}q_{w}+\omega_{y}q_{x}-\omega_{x}q_{y}\right) \end{array}\right]\\ \; & A_{k}=\left[\begin{array}{cc} F_{k} & z_{4x4}\\ z_{4x4} & F_{k} \end{array}\right]\\ \; & q^{d}={\left[\underset{q_{att}}{\underset{︸}{\begin{array}{cccc} q_{0} & q_{1} & q_{2} & q_{3} \end{array}}}\;\underset{q_{hdg}}{\underset{︸}{\begin{array}{cccc} q_{w} & q_{x} & q_{y} & q_{z} \end{array}}}\right]}^{T} \end{array}$$

where $F_{k}$, $z_{4\times 4}$ and $q^{d}$ represent the system matrices used in Equation (A1), zero matrix, and double quaternion states, respectively.

Magnetometer observation model corresponding to heading quaternion:

(A7) $$z=h\left(q_{hdg}\right)=\left[\begin{array}{c} \left(q_{w}^{2}+q_{x}^{2}-q_{y}^{2}-q_{z}^{2}\right)H_{x}+2\left(q_{x}q_{y}+q_{w}q_{z}\right)H_{y}+2\left(q_{x}q_{z}-q_{w}q_{y}\right)H_{z}\\ 2\left(q_{x}q_{y}-q_{w}q_{z}\right)H_{x}+\left(q_{w}^{2}-q_{x}^{2}+q_{y}^{2}-q_{z}^{2}\right)H_{y}+2\left(q_{y}q_{z}+q_{w}q_{x}\right)H_{z}\\ 2\left(q_{x}q_{z}+q_{w}q_{y}\right)H_{x}+2\left(q_{y}q_{z}-q_{w}q_{x}\right)H_{y}+\left(q_{w}^{2}-q_{x}^{2}-q_{y}^{2}+q_{z}^{2}\right)H_{z} \end{array}\right]$$

Lie derivative for double quaternion state representation (i.e., $q=q^{d}$, *n* = 8):

(A8) $$\begin{array}{cc} L_{f}^{0}\left(h\right) & =h(q_{hdg})\\ L_{f}^{1}\left(h\right) & =\frac{\partial }{\partial q^{d}}\left(\begin{array}{c} L_{f}^{0}\left(h\right) \end{array}\right)q_{k}^{d}\\ L_{f}^{2}\left(h\right) & =\frac{\partial }{\partial q^{d}}\left(\begin{array}{c} L_{f}^{1}\left(h\right) \end{array}\right)q_{k}^{d}\\ L_{f}^{3}\left(h\right) & =\frac{\partial }{\partial q^{d}}\left(\begin{array}{c} L_{f}^{2}\left(h\right) \end{array}\right)q_{k}^{d}\\ \vdots \\ L_{f}^{7}\left(h\right) & =\frac{\partial }{\partial q^{d}}\left(\begin{array}{c} L_{f}^{6}\left(h\right) \end{array}\right)q_{k}^{d} \end{array}$$

(A9) $$\begin{array}{cc} O_{2} & =\left[\begin{array}{cccc} \frac{\partial }{\partial q^{d}}\left(\begin{array}{c} L_{f}^{0}\left(h\right) \end{array}\right) & \frac{\partial }{\partial q^{d}}\left(\begin{array}{c} L_{f}^{1}\left(h\right) \end{array}\right) & \frac{\partial }{\partial q^{d}}\left(\begin{array}{c} L_{f}^{2}\left(h\right) \end{array}\right) & \frac{\partial }{\partial q^{d}}\left(\begin{array}{c} L_{f}^{3}\left(h\right) \end{array}\right) \end{array}\right.\\ \; & \;{\left.\begin{array}{cccc} \frac{\partial }{\partial q^{d}}\left(\begin{array}{c} L_{f}^{4}\left(h\right) \end{array}\right) & \frac{\partial }{\partial q^{d}}\left(\begin{array}{c} L_{f}^{5}\left(h\right) \end{array}\right) & \frac{\partial }{\partial q^{d}}\left(\begin{array}{c} L_{f}^{6}\left(h\right) \end{array}\right) & \frac{\partial }{\partial q^{d}}\left(\begin{array}{c} L_{f}^{7}\left(h\right) \end{array}\right) \end{array}\right]}^{T}\\ R_{2} & =rank(O_{2}) \end{array}$$

$O_{2}$ is a 24 × 8 matrix. The rank of $O_{2}$ should be eight for all parameters of the double quaternion (i.e., $q_{0}$, $q_{1}$, $q_{2}$, $q_{3}$, $q_{w}$, $q_{x}$, $q_{y}$ and $q_{z}$) to be observable by the magnetometer sensor.
